# Involvement of CSE/ H_2_S in high glucose induced aberrant secretion of adipokines in 3T3-L1 adipocytes

**DOI:** 10.1186/1476-511X-13-155

**Published:** 2014-10-02

**Authors:** Zhe Pan, Hanbo Wang, Yuantao Liu, Chunxiao Yu, Yuchao Zhang, Jicui Chen, Xiangdong Wang, Qingbo Guan

**Affiliations:** Department of Endocrinology, Shandong Provincial Hospital affiliated to Shandong University, Shandong Clinical Medical Center of Endocrinology and Metabolism; Institute of Endocrinology and Metabolism; Shandong Academy of Clinical Medicine, 324 Jing 5 Rd, Jinan, Shandong 250021 P. R. China; Minimally Invasive Urology Center, Shandong Provincial Hospital affiliated to Shandong University, Jinan, 250021 China; Department of Endocrinology, the Second Hospital of Shandong University, Jinan, 250033 China; Center for Reproductive Medicine, Shandong Provincial Hospital Affiliated to Shandong University; National Research Center for Assisted Reproductive Technology and Reproductive Genetics, Key laboratory for Reproductive Endocrinology of Ministry of Education; Shandong Provincial Key Laboratory of Reproductive Medicine, Jinan, 250012 China; Department of Cell Biology, Shandong University School of Medicine, Jinan, 250012 China

**Keywords:** High glucose, Cystathionine-γ-lyase, H_2_S, Adipokine, Adipocyte

## Abstract

**Background:**

Deregulated secretion of adipokines contributes to subclinical systemic inflammation associated with type 2 diabetes mellitus (T2DM). However, the mechanisms underlying are not fully understood. Hydrogen sulfide (H_2_S), as an endogenous gasotransmitter, possesses an anti-inflammation activity. The aim of this study was to examine the possible involvement of H_2_S in high glucose induced adipokine secretion in 3T3-L1 adipocytes.

**Methods:**

The expression of cystathionine-gamma-lyase (CSE), the H_2_S-forming enzyme, was evaluated by Western-blotting and real-time PCR. The secretion of TNF-α, MCP-1 and adiponectin was determined by radioimmunoassay and enzyme-linked immunosorbent assay (ELISA), respectively. Lentiviral empty vector and vector expressing mouse CSE were used for in vitro transduction.

**Results:**

High glucose (HG) significantly decreased CSE expression at both protein and mRNA levels in mature 3T3-L1 adipocytes. In parallel, HG significantly increased secretion of MCP-1 while decreasing secretion of adiponectin, but had no effect on secretion of TNF-α. HG induced changes in MCP-1 and adiponectin secretion were partly attenuated by forced expression of CSE or sodium hydrosulfide (NaHS), a source of exogenous H_2_S.

**Conclusion:**

High glucose induces aberrant secretion of adipokines in mature 3T3-L1 adipocytes, favoring inflammation. The mechanism is partly related to inhibition of CSE/ H2S system.

## Background

T2DM is now regarded as a low-grade inflammatory disease. Inflammation not only plays an essential role in the development of insulin resistance, but also contributes to the initiation and progression of atherosclerotic lesions in diabetic patients. However, the mechanisms that govern the association between T2DM and the increased synthesis of inflammatory factors are not fully understood.

Adipose tissue, once considered a mere depot for energy storage, is today recognized as an important endocrine organ. Adipocyte is able to secrete a number of factors, collectively referred to as adipokines. Among the well-studied adipokines are a number of metabolic regulators including leptin, resistin and adiponectin, which play important roles in regulation of glucose and lipid metabolism [1, 2]. Other adipokines, such as tumor necrosis factor (TNF-α), interleukin (IL)-6, IL-1β and monocyte chemoattractant protein (MCP)-1, are inflammatory cytokines which are suggested to participate in low-grade pro-inflammatory processes leading to metabolic disorders, insulin resistance, and CVDs [3]. In patients with T2DM, aberrant secretion of adipokines has been suggested to play an important role in the pathogenesis of vascular complications [4].

Hydrogen sulfide (H_2_S) is a lately identified endogenous gasotransmitter. The generation of H_2_S in mammalian tissues is catalyzed by two major enzymes, cystathionine-β-synthase (CBS) and cystathionine-γ-lyase (CSE). Both are pyroxidal-5′-phosphate dependent enzymes that use L-cysteine and homocysteine as substrates [5, 6] A third H_2_S synthesizing enzyme is 3-mercaptopyruvate sulfur transferase (3MST), which utilizes L-cysteine and α-ketoglutarate to produce H_2_S [7]. CSE is the principal H_2_S-forming enzyme in the vasculature and heart, whereas CBS and 3MST are most abundantly expressed in the central nervous system.

Previous studies have shown that H_2_S plays important roles in the physiology and pathophysiology of the cardiovascular, gastrointestinal and neurological systems [8]. In the cardiovascular system, H_2_S acts as a vasorelaxant by opening K + ATP channels [9]. In a myocardial ischemia-reperfusion mouse model, H_2_S was shown to limit infarct size and preserve ventricular functioning [10]. In addition, H_2_S has been shown to have an anti-inflammatory property in many cell types [11, 12]. There has been report showing that cystathionine-γ-lyase (CSE) is also present in adipocytes [13]. However, the precise roles of H_2_S in the physiology and pathophysiology of adipose tissue have not been well documented. In the present study, we investigated the potential role of CSE/ H_2_S system in regulation of adipokine secretion in 3T3-L1 adipocytes under high glucose condition.

## Results

### High glucose downregulated CSE protein expression in 3T3-LI preadipocytes and adipocytes

**Figure 1:**
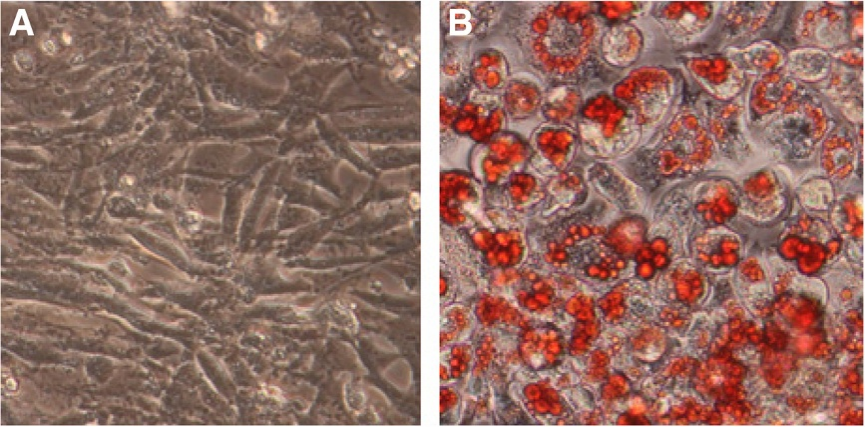
**Adipose differentiation of 3T3-L1 preadipocytes.** Adipose differentiation of 3T3-L1 preadipocytes was induced for 12 days as described in Materials and Methods. Cell differentiation was evaluated by Oil-red-O staining of lipid droplets. **(A)** 3T3-L1 preadipocytes. **(B)** differentiated 3T3-L1 cells. Original magnification 400X.

**Figure 2:**
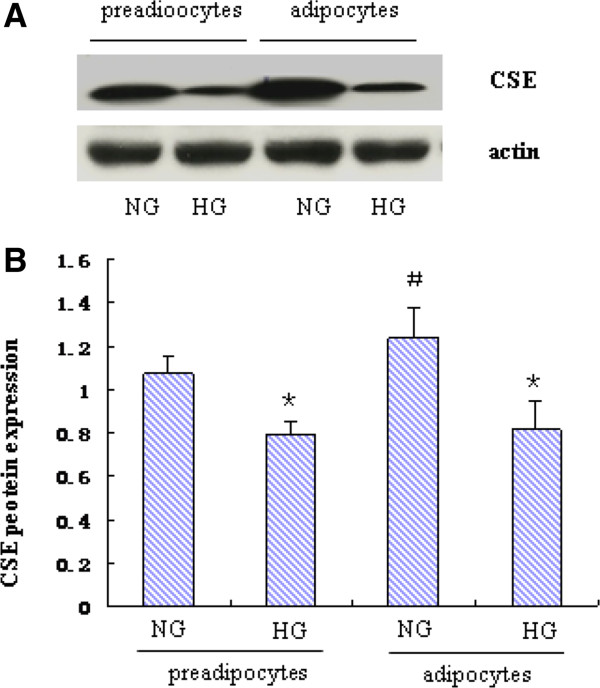
**Effect of high glucose on CSE protein expression in 3T3-LI preadipocytes and adipocytes.** 3T3-LI preadipocytes and adipocytes were treated in normal glucose (5.5 mmol/L) or high glucose (25mmol/L) for 48h. CSE expression was evaluated by Western blotting. **A)** Representative result of Western blots (n = 3). **B)** Quantitative analysis of the results from Western Blots. The value represents the average relative ratio of CSE protein to β-actin in each group (n = 3). ^*^ *P* < 0.05, *vs.* NG, ^#^ *P* < 0.05, *vs*. preadipocytes in NG.

In our previous study, we demonstrated that HG down-regulated CSE expression in HUVEC cells [14]. In the present study, we examined the effect of HG on CSE expression in 3T3-L1 preadipocytes and adipocytes. 3T3-L1 preadipocytes were induced to differentiate as described previously [15]. Cell differentiation was confirmed by Oil-Red-O staining (Figure 1). As shown in Figure 2, CSE expression in differentiated 3T3-L1 adipocytes was significantly high than preadipocytes in normal glucose (5.5 mmol/L). Treatment with HG (25mmol/L) for 48h significantly reduced CSE expression in both 3T3-L1 adipocytes and preadipocytes.

### High glucose reduced CSE mRNA levels in mature 3T3-LI adipocytes

**Figure 3:**
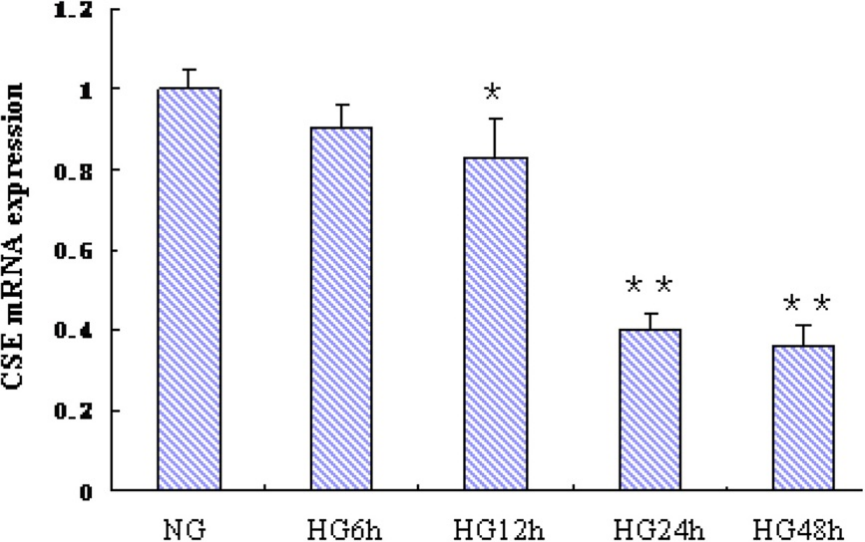
**Effect of high glucose on CSE mRNA expression in mature 3T3-LI adipocytes.** Differentiated 3T3-LI adipocytes were treated in normal glucose (NG, 5.5 mmol/L) or high glucose (HG, 25mmol/L) indicated periods. CSE mRNA expression was evaluated by real time RT-PCR. CSE mRNA level was normalized to GAPDH. Values were expressed as the ratios relative to control (NG). Results are shown as mean ± SD of three independent experiments. ^*^ *P* < 0.05, *vs.* NG; ^**^ *P* < 0.01, *vs.* NG.

The aim of the present study was to explore the pathophysiological role of H_2_S in the mature adipose tissue. Therefore, differentiated 3T3-L1 adipocytes were used in subsequent experiments. To know whether HG regulates CSE expression at mRNA level, we treated 3T3-L1 adipocytes with HG for indicated times. CSE mRNA levels were determined by real-time PCR. As shown in Figure 3, HG treatment significantly reduced CSE mRNA expression as early as 12 h, and the maximum inhibition was observed at 48 h.

### Forced expression of CSE modulated HG induced adipokine secretion profile in mature 3T3-LI adipocytes

**Figure 4:**
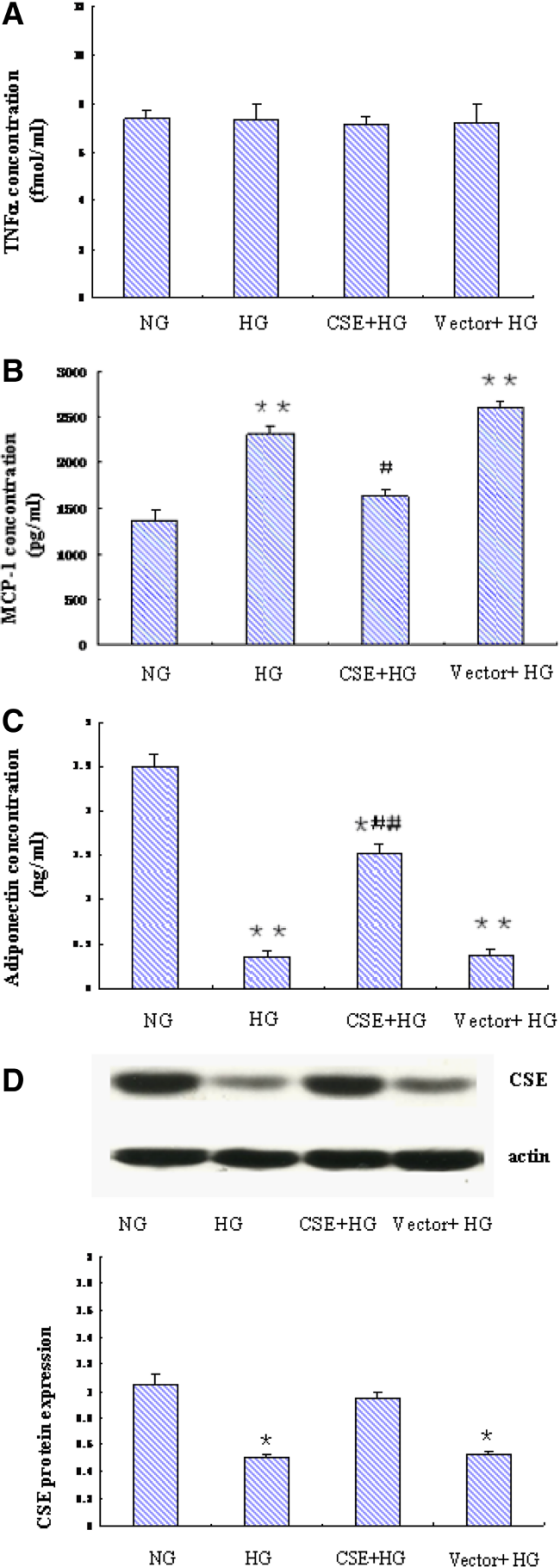
**Effect of forced expression of CSE on the secretion profile of adipokines in mature 3T3-LI adipocytes.** Mature 3T3-LI adipocytes were transfected with lentiviral vector expressing mouse CSE or empty vector for 72h. Transfected and control cells were then treated high glucose (25mmol/L) or normal glucose (5.5mmol/L) for48h. The culture media were then collected for assays of TNFα, MCP-1 and adiponectin **(A, B, C)**. Cell lysates were used for Western blotting analysis of CSE protein expression **(D)**. Results are shown as mean ± SD of three independent experiments. ^*^ *P* < 0.05, ^**^ *P* < 0.01, *vs.* NG alone. ^#^ *P* < 0.05, ^##^ *P* < 0.01, *vs.* HG alone.

H_2_S, the product of CSE, has been shown to have inhibited the secretion of inflammatory factors in other cell types [16, 17]. To determine the potential role of CSE in regulation of adipokine secretion in mature 3T3-LI adipocytes, we over-expressed CSE with a virus vector carrying the CSE gene. The effects of forced expression of CSE were studied. As shown in Figure 4, HG significantly increased secretion of MCP-1, reduced secretion of adiponectin at 24 h and 48 h, but had no evident effect on TNF-α. Fforced expression of CSE significantly attenuated HG induced changes of MCP-1 and adiponectin secretion. The CSE expression was evaluated by Western blotting as shown in Figure 4D.

### NaHS modulated HG induced adipokine secretion profile in mature 3T3-LI adipocytes

**Figure 5:**
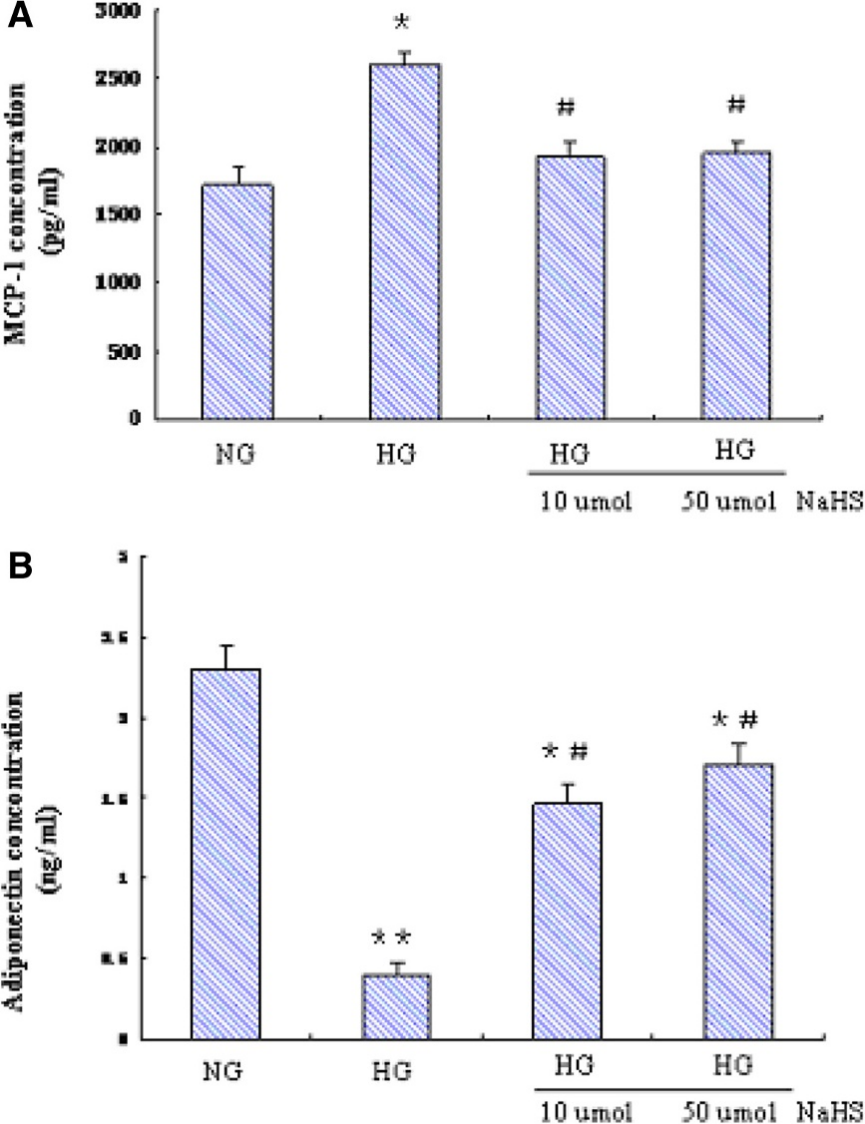
**Effect of NaHS on the secretion profile of adipokines in mature 3T3-LI adipocytes.** Mature 3T3-LI adipocytes were normal glucose (5.5mmol/L) or high glucose (25mmol/L) in the presence or absence of increasing doses of NaHS for 48h. The culture media were then collected for MCP-1 and adiponectin assays **(A, B)**. Results are shown as mean ± SD of three independent experiments. ^*^ *P* < 0.05, ^**^ *P* < 0.01, *vs.* NG alone. ^#^ *P* < 0.05,, ^##^ *P* < 0.01, *vs.* HG alone.

The action of CSE is supposed to be mediated by H_2_S. To confirm a role of H_2_S in regulation of adipokine secretion, we pretreated the cells with NaHS, a commonly used source of exogenous H_2_S, and determined its effects on HG induced adipokine secretion. Similar to forced expression of CSE, NaHS significantly inhibited HG induced MCP-1 secretion while increasing adiponectin secretion (Figure 5).

## Discussion

Clinical studies have showed that levels of inflammatory markers such as C-reactive, IL-6, and TNF-α, are increased in patients with T2DM [18, 19]. Inflammation not only plays an essential role in the development of insulin resistance, but also contributes to the initiation and progression of diabetic vascular complications [20]

It is now well recognized that adipose tissue is an important endocrine organ and a major source of circulating proinflammatory factors such as TNF-α, IL-6, IL-1β and MCP-1 [3, 21]. Adipocyte also secretes numerous other factors such as leptin, resistin and adiponectin, which are important metabolic regulators [1, 2]. Adiponectin is a well-studied adipokine. It is an important insulin-sensitizer that can stimulate glucose uptake in skeletal muscle and reduce hepatic glucose production. In addition, adiponectin also possesses anti-atherogenic and anti-inflammatory properties [22]. So far, the mechanisms leading to increased inflammation in patients with T2DM are not fully understood.

It is well accepted that hyperglycemia is the key risk factor for diabetic vascular complications. The proposed mechanisms whereby high glucose may cause vascular injury include reducing nitric oxide (NO) bioavailability and increasing reactive oxygen species (ROS) leading to endothelial dysfunction [23]. Recent studies showed that glucose can increase the release of proinflammatory cytokines from adipocytes and adipose tissue [24, 25]. However, the signaling mechanisms are unclear. TNF-α, MCP-1 and adiponectin are important factors involved in inflammatory processes. In the present study, we examined the effects of HG on the secretion of these adipokines in mature 3T3-L1 adipocytes. Our results showed that HG significantly increased secretion of MCP-1, reduced secretion of adiponectin, but had no effect on TNF-α secretion. These results suggested that HG differentially regulates the secretion of various adipokines in adipocytes. Our results are consistent with previous studies [24, 25]

H_2_S is a novel endogenous gasotransmitter, which has multiple biological activities such as vasodilation and anti-inflammation [9, 12]. CSE is the principal H_2_S- forming enzyme in the vasculature and heart. Recently, the presence of CSE has been detected in periadventitial adipose tissue [13]. In the present study, we checked the expression of CSE in 3T3-L1 preadipocytes and adipocytes. Our results showed that CSE expression increased after the differentiation of 3T3-L1 preadipocytes. However it is unclear whether CSE plays a role in 3T3-L1 preadipocyte differentiation. Moreover, treatment with HG significantly reduced CSE expression in both 3T3-L1 preadipocytes and adipocytes. The mechanisms by which HG downregulates CSE expression is unknown and need further investigation. In our previous study, we found that CSE regulates the secretion of ICAM-1in endothelial cells [26]. In the present study, we therefore examined the potential role of CSE in regulation of adipokine secretion in mature 3T3-L1 adipocytes. As HG induced aberrant secretion of MCP-1 and adiponectin was accompanied by a reduced CSE expression, we therefore over-expressed CSE and observed its effects. Our results showed that forced expression of CSE significantly attenuated HG induced aberrant secretion of MCP-1 and adiponectin, suggesting that the action of HG on adipokine secretion may at least partly mediated by CSE.

As the function of CSE is supposed to be mediated by H_2_S, we next examined the effects of NaHS, a commonly used source of exogenous H_2_S, on HG induced adipokine secretion. As expected, NaHS significantly inhibited HG induced MCP-1 secretion while increasing adiponectin secretion. To note, HG induced reduction of adiponectin secretion was only partly restored by forced CSE expression or NaHS. It is possible that, in addition to CSE/H_2_S system, HG may also regulate adiponectin secretion through other mechanisms.

In summary, our results showed that high glucose induces aberrant secretion of adipokines favoring inflammation in 3T3-L1 adipocytes, which was partly prevented by forced CSE expression and NaHS administration. These findings suggest that reduced H_2_S production may contribute to deregulated adipokine secretion under high glucose condition. This study provides new insight into the mechanisms underlying T2DM associated inflammation.

## Materials and methods

### Materials

The mouse embryo fibroblasts 3T3-L1 was obtained from the American Type Culture Collection (ATCC). Medium and serum were purchased from GIBCO (Invitrogen, USA). Anti-CSE antibody was from Thermo Scientific (IL, USA); Anti-β-actin antibody was from Santa Cruz (Cambridge, UK). All primers used in this study were synthesized in Genomics Institute of Sangon Biotech in Shanghai. All other reagents were purchased from standard suppliers.

### Cell culture

The preadipoctyes were maintained in Dulbecco’s modified Eagle’s medium (DMEM, Gibco BRL) supplemented with 10% (v/v) newborn calf serum(NBS), 100 U/mL penicillin, and 0.1 mg/mL streptomycin (KeyGEN, Nanjing, China) in a humidified atmosphere composed of 95% air and 5% CO_2_ at 37°C . Cell differentiation was induced as described previously [15]. In brief, confluent preadipocytes were treated for 2 days with insulin (10 μM), isobutylmethylxanthine (IBMX) (0.5 mM), and dexamethasone (0.25 μM) in DMEM containing 10% FCS, followed by treatment for another 2 days with insulin (10 μM) alone in DMEM containing 10% FCS. Afterwards, cells were replenished with DMEM containing 10% FCS every other day. On day 12, more than 90% cells were differentiated into mature adipocytes as confirmed by Oil-Red-O staining [15].

### Cell treatment

Cells were grown to approximately 80% confluence in 60mm cell culture dishes, the medium was then changed to M199 medium supplemented with 2% FBS and 100U/mL penicillin-streptomycin. Cells were then cultured in medium containing either normal glucose (5.5 mmol/L) which served as a normal control or high glucose (25mmol/L) for indicated periods. For experiments with NaHS, cells were pre-treated with NaHS (10 μmol/L or 50 μmol/L) for 30 min prior to other treatments. Mannitol (24.5 mmol/L) was always added to medium containing normal glucose (5.5 mmol/L) to make osmotic pressure equal to high glucose unless otherwise stated.

### In vitro transduction

Lentiviral empty vector and vector expressing mouse CSE were provided Genepharma (Shanghai, China). Lentiviral plasmids were transfected into HEK293 cells to generate the lentiviruses. Mature 3T3-LI adipocytes were infected with the lentiviral vectors (1 × 10^9^ transducing units [TU]/ml) for 72 h. CSE protein level was measured by western blotting as indicated.

### Western blot analysis

Cells were homogenized in RIPA lysis buffer containing protease and phosphatase inhibitors. Protein concentration was determined by Bradford method. Equal amounts of protein from cell lysates were loaded in each well of a 10% sodium dodecyl sulfate–polyacrylamide gel. After electrophoresis, proteins were transferred to polyvinylidene fluoride membranes, blocked for 1 hour with 5% fat-free milk at room temperature, and blotted with primary antibodies (anti-CSE antibody, 1:1000; anti-β-actin antibody, 1:2000) overnight at 4°C with gentle agitation. After washing with Tris-buffered saline tween-20 (TBST),the membranes were incubated with horseradish peroxidase-conjugated secondary antibodies for 1 hour at room temperature. Then, immune complexes were detected using the enhanced chemiluminescence system (SuperSignal West Pico Chemiluminescent Substrate, Pierce), and immunoreactive bands were quantified using Image J software.

### Real-time PCR

Total RNA was extracted from adipose cell using Trizol (TaKaRa) according to manufacturer’s instruction. Total RNA (4 ug) was used as a template for RT with the ReverTra Ace® qPCR RT kit (TOYOBO). Amplification was performed using the CFX96TM Real-Time System (Bio-Rad) at 95°C for 3 minutes, followed by 40 cycles of 95°C for 10seconds and 55°C for 30 seconds and 72°C for 1 minutes using the following primers: b-actin: 5-GTGACGTTGACATCCGTAAAGA-3′ (forward), R:5-GCCGGACTCATCGTACTCC-3′ (reverse); CSE: -CCATCCACGTGGGACAAGAG-3′(forward), R:5-GCGGCTGTATTCAAAACCCG-3′ (reverse). The results of the real-time PCR data were expressed as Cq values, where Cq can be defined as the threshold cycle of PCR at which amplified PCR product was first detected. The relative amount of CSE mRNA was normalized to the housekeeping gene, β-actin. The relative mRNA expression level was expressed as fold change over control as determined by the 2-ΔΔ Cq method. The average of the relative amount of mRNA in control group is defined as 1.0. The factor X by which the amount of the changed gene can be calculated with the following formula: X = 2-ΔΔ Cq, where ΔΔ Cq = (Cq target gene − Cq β − actin ) sample − (Cqtarget gene − Cq β-actin ) control.

### Enzyme-linked immunosorbent assay for MCP-1 and adiponectin

After treatments, the cell culture medium was collected and centrifuged (12,000 rotations/min) for 20 min to remove debris. The supernatants were collected for MCP-1 and adiponectin measurements using enzyme-linked immunosorbent assay (ELISA) kits (CASABIO, Wuhan, China) according to the manufacturer’s instructions.

### TNF-α assay

After treatments, the cell culture medium was collected and centrifuged (12,000 rotations/min) for 20 min to remove debris. The supernatants were collected for TNF-α measurement using ^125^I TNF Radioimmunoassay Kit (Beijing North Institute of Biological Technology, Beijing, China) according to the manufacturer’s instructions.

### Statistical analysis

Experimental data are presented as the Mean ± SE. Differences between groups were examined by one-way ANOVA followed by Newman-Keuls tests using SPSS16.0 software. A value of P < 0.05 was considered statistically significant.

## References

[1] Ouchi N, Parker JL, Lugus JJ, Walsh K (2011). Adipokines in inflammation and metabolic disease. Nat Rev Immunol.

[2] Trujillo ME, Scherer PE (2006). Adipose tissue–derived factors: impact on health and disease. Endocr Rev.

[3] Bahceci M, Gokalp D, Bahceci S, Tuzcu A, Atmaca S, Arikan S (2007). The correlation between adiposity and adiponectin, tumor necrosis factor alpha, interleukin-6 and high sensitivity C-reactive protein levels. Is adipocyte size associated with inflammation in adults?. J Endocrinol Invest.

[4] Cesari M, Penninx BW, Newman AB, Kritchevsky SB, Nicklas BJ, Sutton-Tyrrell K, Rubin SM, Ding J, Simonsick EM, Harris TB, Pahor M (2003). Inflammatory markers and onset of cardiovascular events: results from the health ABC study. Circulation.

[5] Wang R (2002). Two’s company, three’s a crowd: can H2S be the third endogenous gaseous transmitter?. FASEB J.

[6] Kimura H (2011). Hydrogen sulfide: its production, release and functions. Amino Acids.

[7] Shibuya N, Tanaka M, Yoshida M, Ogasawara Y, Togawa T, Ishii K, Kimura H (2009). 3-mercaptopyruvate sulfur transferase produces hydrogen sulfide and bound sulfane sulfur in the brain. Antioxid Redox Signal.

[8] Hu LF, Lu M, Hon Wong PT, Bian JS (2008). Hydrogen sulfide: neurophysiology and neuropathology. Antioxid Redox Signal.

[9] Yang G, Wu L, Jiang B, Yang W, Qi J, Cao K, Meng Q, Mustafa AK, Mu W, Zhang S, Snyder SH, Wang R (2008). H2S as a physiologic vasorelaxant: hypertension in mice with deletion of cystathionine-sigma -lyase. Science.

[10] Elrod JW, Calvert JW, Morrison J, Doeller JE, Kraus DW, Tao L, Jiao X, Scalia R, Kiss L, Szabo C, Kimura H, Chow CW, Lefer DJ (2007). Hydrogen sulfide attenuates myocardial ischemia-reperfusion injury by preservation of mitochondrial function. Proc Natl Acad Sci U S A.

[11] Szabo C (2007). Hydrogen sulphide and its therapeutic potential. Nat Rev Drug Discov.

[12] Oh GS, Pae HO, Lee BS, Kim BN, Kim JM, Kim HR, Jeon SB, Jeon WK, Chae HJ, Chung HT (2006). Hydrogen sulfide inhibits nitric oxide production and nuclear factor-kB via heme oxygenase-1 expression in RAW264.7 macrophages stimulated with lipopolysaccharide. Free Radic Biol Med.

[13] Fang L, Zhao J, Chen Y, Ma T, Xu G, Tang C, Liu X, Geng B (2009). Hydrogen sulfide derived from periadventitial adipose tissue is a vasodilator. J Hypertens.

[14] Guan Q, Zhang Y, Yu C, Liu Y, Gao L, Zhao J (2012). Hydrogen sulfide protects against high-glucose-induced apoptosis in endothelial cells. J Cardiovasc Pharmacol.

[15] Lu S, Guan Q, Liu Y, Wang H, Xu W, Li X, Fu Y, Gao L, Zhao J, Wang X (2012). Role of extrathyroidal TSHR expression in adipocyte differentiation and its association with obesity. Lipids Health Dis.

[16] Song K, Wang F, Li Q, Shi YB, Zheng HF, Peng H, Shen HY, Liu CF, Hu LF (2014). Hydrogen sulfide inhibits the renal fibrosis of obstructive nephropathy. Kidney Int.

[17] Tamizhselvi R1, Sun J, Koh YH, Bhatia M (2009). Effect of hydrogen sulfide on the phosphatidylinositol 3-kinase-protein kinase B pathway and on caerulein-induced cytokine production in isolated mouse pancreatic acinar cells. J Pharmacol Exp Ther.

[18] de Rekeneire N, Peila R, Ding J, Colbert LH, Visser M, Shorr RI, Kritchevsky SB, Kuller LH, Strotmeyer ES, Schwartz AV, Vellas B, Harris TB (2006). Diabetes, hyperglycemia, and inflammation in older individuals: the health, aging and body composition study. Diabetes Care.

[19] Nilsson J, Jovinge S, Niemann A, Reneland R, Lithell H (1998). Relation between plasma tumor necrosis factor-and insulin sensitivity in elderly men with noninsulin-dependent diabetes mellitus. Arterioscler Thromb Vasc Biol.

[20] Li ZY, Wang P, Miao CY (2011). Adipokines in inflammation, insulin resistance and cardiovascular disease. Clin Exp Pharmacol Physiol.

[21] Mohamed-Ali V, Goodrick S, Rawesh A, Katz DR, Miles JM, Yudkin JS, Klein S, Coppack SW (1997). Subcutaneous adipose tissue releases interleukin-6, but not tumor necrosis factor-α, in vivo. J Clin Endocrinol Metab.

[22] Choi SH, Hong ES, Lim S (2013). Clinical implications of adipocytokines and newly emerging metabolic factors with relation to insulin resistance and cardiovascular health. Front Endocrinol (Lausanne).

[23] Creager MA, Luscher TF, Cosentino F, Beckman JA (2003). Diabetes and vascular disease: pathophysiology, clinical consequences, and medical therapy: part I. Circulation.

[24] Sun J, Xu Y, Dai Z, Sun Y (2009). Intermittent high glucose stimulate MCP-l, IL-18, and PAI-1, but inhibit adiponectin expression and secretion in adipocytes dependent of ROS. Cell Biochem Biophys.

[25] He G1, Bruun JM, Lihn AS, Pedersen SB, Richelsen B (2003). Stimulation of PAI-1 and adipokines by glucose in human adipose tissue in vitro. Biochem Biophys Res Commun.

[26] Guan Q, Wang X, Gao L, Chen J, Liu Y, Yu C, Zhang N, Zhang X, Zhao J (2013). Hydrogen sulfide suppresses high glucose-induced expression of intercellular adhesion molecule-1 (ICAM-1) in endothelial cells. J Cardiovasc Pharmacol.

